# Isolation and Characterization of ΦCA1NRNZ, a Lytic Bacteriophage Targeting the Emerging Device-Associated Pathogen *Cutibacterium avidum*

**DOI:** 10.3390/antibiotics15070659

**Published:** 2026-07-03

**Authors:** Ron Braunstein, Amit Rimon, Roni Teitelbaum, Suhnit Coppenhagen-Glazer, Vered Molho-Pessach, Ronen Hazan

**Affiliations:** 1 Institute of Biomedical and Oral Research (IBOR), Faculty of Dental Medicine, The Hebrew University of Jerusalem, Jerusalem 9112000, Israel; ron.brnstn@gmail.com (R.B.); amit.rimon@mail.huji.ac.il (A.R.); roni9312@gmail.com (R.T.); tinushy@yahoo.com (S.C.-G.); 2 The Israeli Phage Center (IPTC) of the Hebrew University and Hadassah Medical Center, Jerusalem 9112000, Israel; 3 Tzameret, The Military Track of Medicine, The Hebrew University-Hadassah Medical School, Jerusalem 9112000, Israel; 4 Department of Dermatology, Hadassah Medical Center, The Faculty of Medicine, The Hebrew University of Jerusalem, Jerusalem 9112000, Israel; rverem@hadassah.org.il

**Keywords:** *Cutibacterium avidum*, phage therapy, biofilm inhibition

## Abstract

**Background**: *Cutibacterium avidum* is an emerging opportunistic pathogen responsible for device-associated infections, including prosthetic joint and breast implant infections. Unlike its relative *C. acnes*, for which phage therapy has been explored, *C. avidum* infections are recalcitrant to antibiotics, and no infecting bacteriophages have been described to date. Here, we report the isolation and characterization of ΦCA1NRNZ, to the best of our knowledge, the first lytic phage described against *C. avidum*. **Methods**: ΦCA1NRNZ was obtained from wastewater sampling at the Sorek Treatment Facility in Jerusalem. Wastewater metagenomics, transmission electron microscopy, genome sequencing, host-range testing, efficiency of plating (EOP), aerobic and anaerobic lysis assays, and antibiofilm assays against mature *C. avidum* biofilms were performed. **Results**: Metagenomic analysis indicated low and transient detection of *C. avidum*-classified reads in wastewater. ΦCA1NRNZ was identified as a long-tailed Caudoviricetes with a ~320 nm virion. Its 33,712 bp dsDNA genome (GenBank PV441878.1) encodes 46 predicted proteins, shares 76.5% nucleotide identity with *C. acnes* phage ΦFD1, and contains divergent tail-fiber and host-recognition genes. No known bacterial virulence, toxin, human pathogenicity-associated, or antibiotic-resistance genes were identified. ΦCA1NRNZ lysed all 11 clinical *C. avidum* isolates tested under aerobic and anaerobic conditions, with EOP values of 0.11–5.55, mean 1.87, and showed no lytic activity against 25 *C. acnes* isolates. Against mature biofilms, ΦCA1NRNZ reduced total biomass by 28.4% (*p* = 0.014), reduced viable cell counts by approximately two logs, and increased extracellular ATP release (*p* < 0.001). **Conclusions**: The strict species specificity and significant in vitro antibiofilm activity of ΦCA1NRNZ support its potential for phage therapy of device-associated *C. avidum* infections.

## 1. Introduction

*Cutibacterium avidum*, formerly *Propionibacterium avidum*, is a skin commensal increasingly recognized as an opportunistic pathogen [1,2]. It has been implicated in diverse infections, particularly in immunocompromised patients and those with implanted medical devices such as prosthetic joints and breast implants [2,3,4]. Its slow growth and biofilm-forming capacity contribute to delayed diagnosis, chronicity, and treatment challenges [5]. Recent evidence also links *C. avidum* to acne vulgaris, expanding the traditional focus beyond *Cutibacterium acnes* in a disease affecting ~80% of adolescents and young adults worldwide [6]. The average nucleotide identity (ANI) between *C. acnes* and *C. avidum* is approximately 88% [1].

Bacteriophages, viruses that specifically infect bacteria, are re-emerging as promising therapeutics amid the global antibiotic resistance crisis [7,8]. They can target killing, bypass antibiotic resistance, and disrupt biofilms. To the best of our knowledge, no phages infecting *C. avidum* have been described to date, whereas phages infecting *C. acnes*, a related *Cutibacterium* species, have been extensively studied for their potential in acne treatment, including in our previous work [9,10,11]. Notably, interest in such phages has grown, as illustrated by a request from Phage Directory (15.12.20) for compassionate treatment of a patient infected with a *C. avidum* clinical isolate (https://phage.directory/alerts, “alert from December 15, 2020”, accessed on 1 July 2026). 

Previously, we demonstrated that topical phage therapy targeting *C. acnes* can significantly reduce acne-like inflammation in mice, suggesting its promise as a novel approach to treat antibiotic-resistant infections [12].

Here, we report, to the best of our knowledge, the first isolation and characterization of a *C. avidum* infecting phage, termed ΦCA1NRNZ. We describe its isolation, morphology, and genome, assess its host range across *C. avidum* clinical isolates, and evaluate its antibiofilm activity. Because *C. avidum* and *C. acnes* are phylogenetically related and occupy overlapping skin-associated niches, we also tested whether ΦCA1NRNZ could infect *C. acnes* clinical isolates and whether its host range was species-restricted. This work addresses a gap in phage therapy for anaerobic pathogens. It may inform novel strategies for managing *C. avidum* infections and future phage-based approaches for skin- and device-associated infections.

## 2. Results

Wastewater samples were selected as a source for phage isolation because they are recognized reservoirs of diverse environmental and human-associated bacteriophages and have previously been used to isolate phages targeting related *Cutibacterium* species [11,12,13]. Phage ΦCA1NRNZ was isolated from the ‘First Treatment’ sampling point at the Sorek Wastewater Treatment Facility (Jerusalem, Israel), using *Cutibacterium avidum* strain 48 as the isolation host. This sampling point corresponds to an early primary settling stage at the beginning of the wastewater treatment process, upstream of the Bio-Anaerobic and Bio-Aerobic biological treatment steps. At this stage, incoming wastewater undergoes gravitational settling, allowing heavy particulate material to sediment before biological processing.

For phage isolation, wastewater from this sampling point was clarified by centrifugation and filtration, and the filtrate was enriched with *C. avidum* strain 48. Following enrichment, the culture was filtered again, and the resulting filtrate was spotted onto a bacterial lawn of *C. avidum* 48, where plaque-forming activity was observed. The initial plaque-positive area was sampled and transferred onto a fresh *C. avidum* 48 lawn to obtain isolated plaques. A well-isolated plaque was then picked and subjected to five consecutive rounds of plaque purification on fresh *C. avidum* 48 lawns. Reproducible plaque formation throughout these passages indicated that the observed clearing resulted from the propagation of an infective phage rather than from non-replicative antimicrobial activity in the wastewater filtrate. Following serial plaque purification, a purified plaque isolate was amplified on *C. avidum* 48 and designated ΦCA1NRNZ. Its identity as a bacteriophage was further supported by transmission electron microscopy and whole-genome sequencing, as described below.

To examine the distribution of *C. avidum* DNA across the wastewater-treatment process and its relation to the isolation source, we performed metagenomic sequencing of samples taken sequentially across five treatment stages of the Sorek facility (Start Point, First Treatment, Bio-Anaerobic, Bio-Aerobic, and Before HCl) and classified reads using Kraken2/Bracken. Because *C. avidum* is a human-associated bacterium, detection of *C. avidum*-classified reads in wastewater provided a rationale for targeting these samples for *C. avidum* phage isolation, given that phages are generally expected to be recovered from environments in which their bacterial host is present.

**Figure 1:**
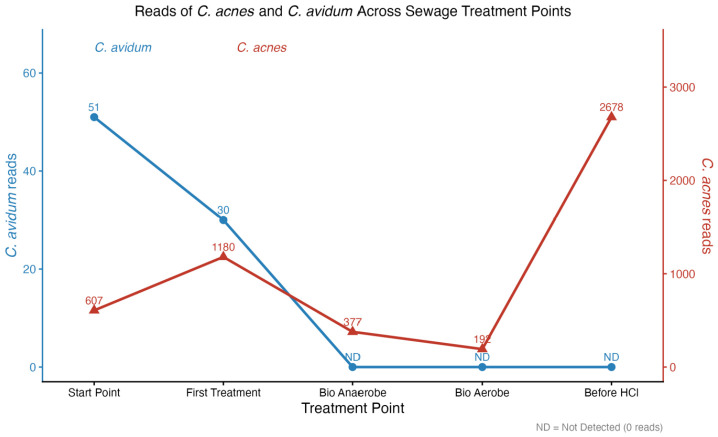
Wastewater metagenomics. Read counts of *Cutibacterium avidum* (left axis, blue) and *Cutibacterium acnes* (right axis, red) across five sequential wastewater treatment points at the Sorek Wastewater Treatment Facility: Start Point, First Treatment, Bio Anaerobe, Bio Aerobe and Before HCl. Values represent species-level Kraken2/Bracken-classified read counts from metagenomic sequencing of five sampling points. *C. avidum* classified-reads dropped from 51 to 30 and were undetected (ND, 0 reads) from Bio Anaerobe onward, while *C. acnes* remained detectable at all points (607–2678 reads). Together, these data indicate low and transient detection of *C. avidum* in early wastewater treatment stages, consistent with the need for multiple independent sampling rounds before a *C. avidum*-infecting phage was recovered.

*C. avidum*-classified reads were detected only at the two earliest treatment points, Start Point and First Treatment (51 and 30 reads, respectively), and were not detected in any subsequent stages (points 3–5), consistent with low and transient detection of *C. avidum*-classified reads in early-wastewater treatment stages (Figure 1). This pattern was compared with *C. acnes*, which served here as an environmental reference organism: *C. acnes* was consistently detected across all five treatment points (607, 1180, 377, 192, and 2678 reads at points 1–5, respectively), suggesting a broader and more consistent detection pattern for this closely related species across the treatment process. Together, these findings provide a rationale for targeting early wastewater-treatment samples for *C. avidum* phage isolation.

### 2.1. Genome Analysis

**Figure 2:**
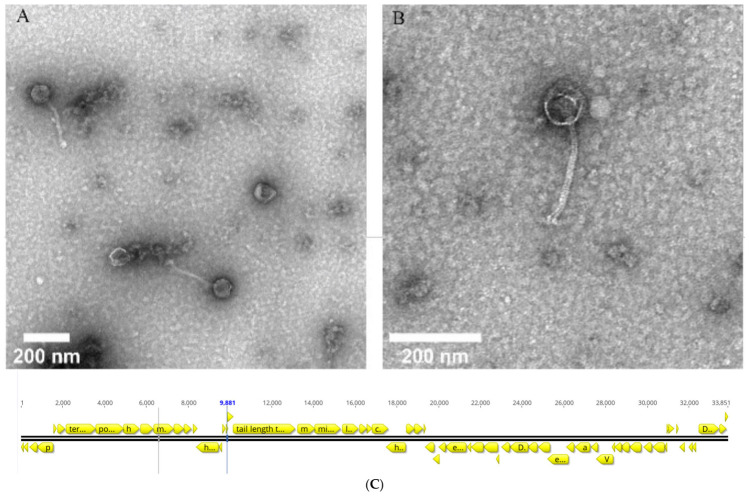
Phage morphology and genome organization. (**A**,**B**) Transmission electron microscopy (TEM) images of the isolated phage. (**C**) Visualization of the CA1NRNZ phage genome with annotated features, generated using Geneious Prime v2024.0.3.

ΦCA1NRNZ is a long-tailed phage with an icosahedral capsid measuring approximately 320 nm (Figure 2A,B). The phage’s genome is a 33,712 bp double-stranded DNA (dsDNA) molecule (accession number PV441878.1). It is classified in the *Caudoviricetes* class, currently unassigned to any genus or family, with genomic similarity to members of the genus *Pahexavirus* but insufficient to meet the 70% ICTV threshold for assignment to that genus [13,14]. It has lytic lifestyle characteristics as detected by PhaBox v2.1.13 (https://phage.ee.cityu.edu.hk/, accessed on 4 June 2026). PhageTerm classification showed that ΦCA1NRNZ has a prominent forward-strand terminus at position 33,558, with redundant and partially permuted ends consistent with headful packaging and linear genome structure. Analysis of virulence factors using abricate (https://github.com/tseemann/abricate release 14, accessed on 4 June 2026) did not identify known bacterial virulence, toxin, human pathogenicity-associated, or antibiotic-resistance genes.

**Figure 3:**
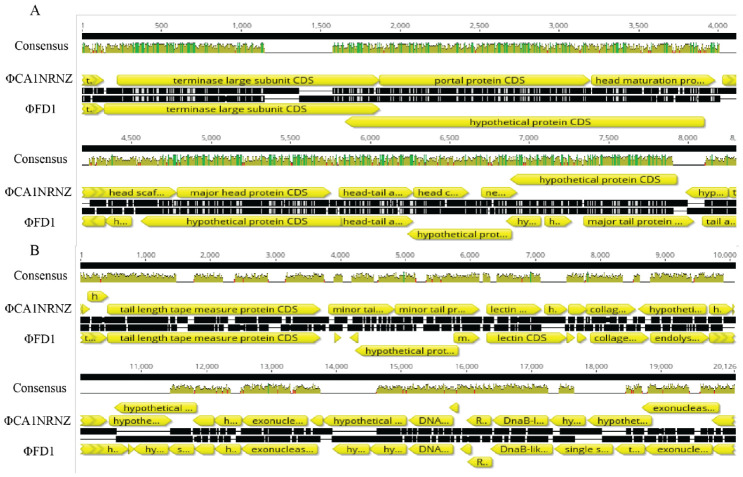
Whole-genome alignment of *Cutibacterium* phages ΦFD1 and ΦCA1NRNZ. Syntenic comparison of the genomes of ΦFD1, a *Cutibacterium* phage, and ΦCA1NRNZ, using Mauve whole-genome alignment. Consensus regions of sequence conservation are highlighted, with the alignment algorithm dividing the genomic comparison into Locally Collinear Block 1 (LCB1; (**A**)) and LCB2 (**B**).

Genomic annotation identified 46 predicted protein-coding genes (Figure 2C). These include key structural components such as the major head protein, portal protein, tail length tape measure protein, and terminase subunits, consistent with the organization of a tailed bacteriophage. The presence of DNA replication and repair genes, including a DnaB-like helicase (XRX15559.1), DNA primase (XRX15557.1), exonucleases (XRX15554.1, XRX15562.1), and a Holliday junction resolvase (XRX15558.1), suggests that the phage encodes its own replication machinery. Additionally, genes for an anti-restriction protein (XRX15564.1) and a predicted phage fitness factor (XRX15565.1) suggest mechanisms to help the phage evade bacterial defenses. Nearly half of the predicted proteins remain hypothetical, underscoring the genetic novelty of this phage. We compared the ΦCA1NRNZ genome (accession number PV441878.1) to the *Cutibacterium acnes* phage ΦFD1 (accession number MW161461.1), as a representative of the *Pahexavirus* genus (class Caudoviricetes, phylum Uroviricota) infecting *C. acnes*, given the known high similarity among these phages [9,12]. Both phages encode dsDNA genomes with a similar overall architecture, and whole-genome BLAST+ (version 2.17.0) alignment revealed a nucleotide identity of 76.54%, indicating close evolutionary proximity. Comparative annotation of the locally collinear block shared between ΦCA1NRNZ and ΦFD1 shows strong conservation of the core structural and functional backbone, with contiguous, similarly positioned genes for virion assembly and genome packaging (including head closure, head maturation protease, head scaffolding, head-tail adaptor, major head/tail, neck, portal, and terminase large and small subunits), as well as several hypothetical proteins of comparable size, position, and orientation. In contrast, regions encoding host-recognition elements, particularly the minor tail and tail assembly genes, show pronounced sequence and orientation divergence, suggesting variability in host-interaction modules despite the otherwise conserved genome backbone. Full coordinate-level comparisons for all homologous genes are provided in Supplementary Table S1 (Figure 3).

### 2.2. Assessment of Antimicrobial Activity

**Figure 4:**
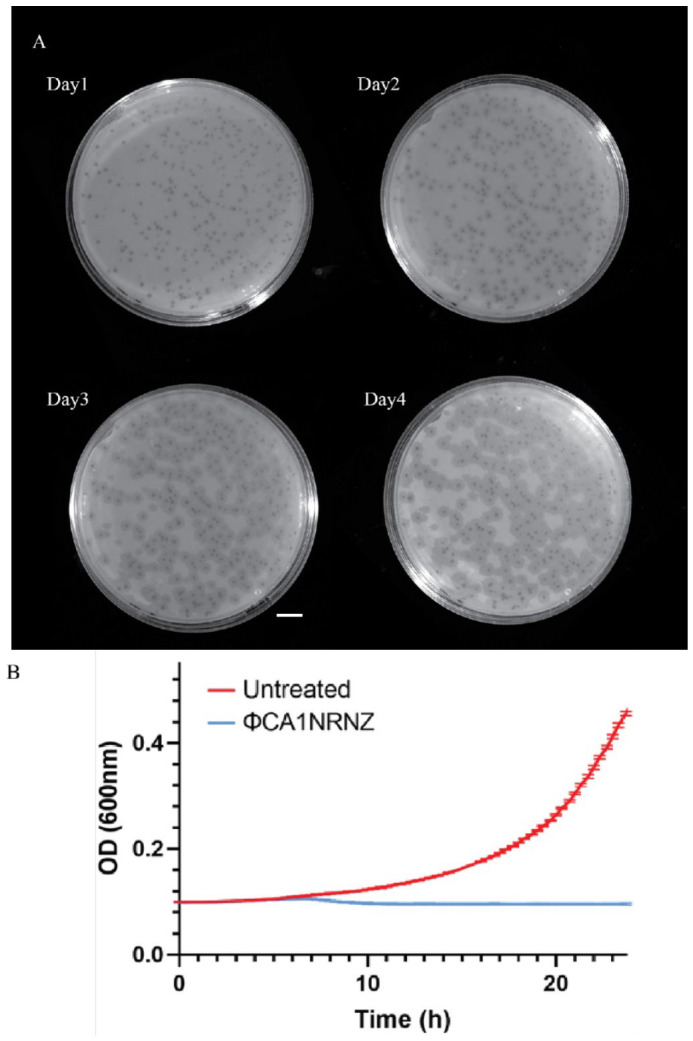
ΦCA1NRNZ plaque morphology and activity in liquid medium. (**A**) Plaque morphology of ΦCA1NRNZ over 4 days on *C. avidum* 48 bacterial lawns. ΦCA1NRNZ visible halo expansion. (**B**) *C. avidum* 48 growth kinetics treated with ΦCA1NRNZ in anaerobic conditions. Data are presented as the mean values obtained from three independent experiments, with error bars indicating the standard deviation (±SD).

**Figure 5:**
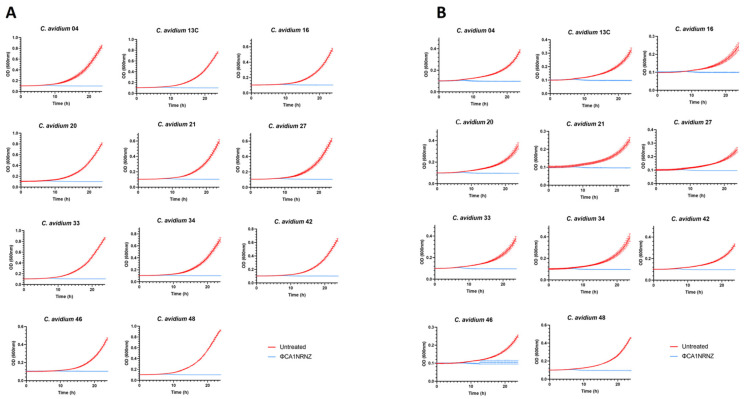
Assessment of ΦCA1NRNZ activity in liquid medium. The phage was tested against 11 *C. avidum* strains at an MOI of 1 under aerobic conditions (**A**) and anaerobic conditions (**B**). No recovery of bacterial growth was observed in ΦCA1NRNZ-treated cultures during the 24 h monitoring period. Data are presented as the mean values obtained from three independent experiments, with error bars indicating the standard deviation (±SD).

To evaluate the lytic activity of ΦCA1NRNZ against *C. avidum*, we tested the phage on 11 clinical isolates using the Israeli Phage Therapy Center’s standard protocol [8]. ΦCA1NRNZ exhibited consistent plaque morphology across all tested strains, forming clear, centrally located plaques with surrounding turbid halos (Figure 4). Plaques continued to expand for at least 96 h post-inoculation. Compared to the phage titer on its original host, *C. avidum* 48, the phage demonstrated variable but generally high efficiency of plating (EOP), with values ranging from 0.11 to 5.55 (mean EOP = 1.87), indicating robust lytic activity across all isolates and resulting in a “susceptible” score interpretation for each strain (Supplementary Table S2). To further characterize the lytic activity of ΦCA1NRNZ in suspension, growth kinetics were monitored over 24 h in liquid medium at a target MOI of 1, under both aerobic (Figure 5A) and anaerobic (Figure 5B) conditions. In untreated controls, all 11 isolates exhibited growth, regardless of oxygen conditions.

In contrast, ΦCA1NRNZ-treated cultures remained suppressed at near-baseline OD600 nm (0.10–0.117 corresponding to ~10–20% of the untreated, which was ~0.6–1.0) throughout the full 24 h observation window in both aerobic and anaerobic conditions, with no visible recovery of bacterial growth across any of the tested strains (Figure 5). This sustained growth inhibition in liquid medium is consistent with the broad lytic activity observed in plaque assays. It confirms that ΦCA1NRNZ maintains its bactericidal efficacy in suspension across the range of oxygen tensions relevant to *C. avidum* clinical infections, which occur in both aerobic tissue environments and oxygen-depleted device-associated niches.

**Figure 6:**
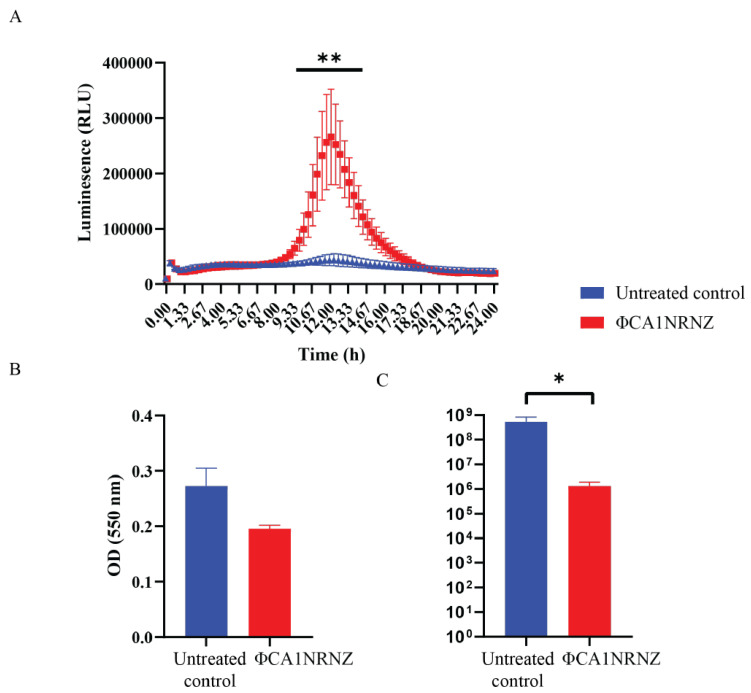
Assessment of phage activity against biofilms. Analysis of phage activity against biofilms using three distinct methods. (**A**) Extracellular ATP release was measured as an indicator of bacterial lysis, reflecting phage-induced disruption of biofilm integrity. (**B**) Biomass was quantified using the crystal violet assay, providing insights into biofilm mass reduction. (**C**) Post-sonication colony-forming unit (CFU) analysis assessed bacterial viability by enumerating surviving cells after phage treatment. Together, these complementary approaches offer a comprehensive evaluation of phage efficacy in targeting biofilm-associated bacteria. Data are presented as the mean values obtained from three independent experiments, with error bars indicating the standard deviation (±SD). Statistical significance was determined using a two-tailed Student’s *t*-test; for panel (**C**), statistical testing was performed on log10-transformed CFU/mL values. *p*-values < 0.05 considered statistically significant (*, *p* < 0.05; **, *p* < 0.001).

ΦCA1NRNZ was also evaluated for its antibiofilm activity against mature *C. avidum* 48 h biofilms. Lysis, as measured by extracellular ATP release, began approximately 8 h post-treatment and peaked at 12 h with a relative luminescence value (RLU) of 266,000 ± 70,304, followed by a gradual decline (Figure 6A, *p*-value < 0.001). The mean Crystal Violet decreased from 0.273 ± 0.032 in the untreated control group to 0.195 ± 0.006 in the phage-treated group, reflecting a 28.4% reduction (Figure 6B, *p*-value = 0.014), and viable cell counts showed about a 2-log decrease, demonstrating the phage’s antibiofilm potential (Figure 6C, *p*-value < 0.05).

As a member of the *Caudoviricetes* class, and due to the close phylogenetic relationship between *C. avidum* and *C. acnes*, ΦCA1NRNZ was also tested against 25 clinical isolates of *C. acnes* using plaque assays. However, all strains were resistant, and no lytic activity was observed.

## 3. Discussion

In this study, we characterize ΦCA1NRNZ, a lytic phage that infects *Cutibacterium avidum*. To the best of our knowledge, this is the first report of the isolation and characterization of a *C. avidum*-infecting phage.

ΦCA1NRNZ belongs to the class *Caudoviricetes*, and it is closely related to members of the *Pahexavirus* genus, a small phage family that only infects *Cutibacterium acnes* [10]. *C. avidum* and *C. acnes* are closely related Gram-positive actinobacteria that inhabit human skin, particularly sebaceous regions [13]. They share significant genomic and metabolic features, including lipid metabolism and propionic acid fermentation [13], and phylogenetic analyses based on 16S *rRNA* and core genomes support their relatedness within the *Cutibacterium* genus, with average nucleotide identity (ANI) values above 85% [13,14,15,16]. In line with this relationship, ΦCA1NRNZ shares 76% DNA sequence identity with known *C. acnes* phages, suggesting genomic relatedness between phages infecting these two *Cutibacterium* species. However, no cross-infection was observed: the tested *C. acnes* phages did not infect any *C. avidum* strains, and ΦCA1NRNZ did not infect any of the tested *C. acnes* strains.

Comparative analysis of the annotated gene content of ΦCA1NRNZ and the *C. acnes*-targeting phage ΦFD1 indicates that both phages share a conserved genomic backbone of essential structural modules, reflecting close evolutionary relatedness between them. The presence of several homologous hypothetical genes in similar loci further suggests functional similarities that remain to be characterized. In contrast, variation in minor tail and tail-associated genes likely explains the distinct host ranges and infection specificities observed in these phages [17]. These divergent tail-associated regions are therefore potential molecular determinants of host interaction and provide targets for future studies of phage-host specificity in *Cutibacterium*.

*C. avidum* has emerged in recent years as an opportunistic pathogen associated with a variety of infections. Notably, it has been implicated in prosthetic joint infections, particularly following hip arthroplasty [2], as well as in breast implant and breast abscess infections [3]. In parallel, phage therapy has regained interest as a promising strategy to combat persistent bacterial infections, particularly those involving antibiotic-resistant or biofilm-forming strains [7]. A key limitation of phage therapy is the typically narrow host range of most phages, which often requires pathogen-specific matching [8]. In our CPM screening protocol, ΦCA1NRNZ showed a strong lytic effect against a broad panel of clinical *C. avidum* isolates in both plaque and liquid assays, with comparable efficacy under aerobic and anaerobic conditions, consistent with the aerotolerant anaerobic nature of this bacterium [1]. To evaluate its antibiofilm potential, we performed three complementary assays measuring bacterial lysis, total biomass, and viable cell counts. Across these assays, ΦCA1NRNZ significantly reduced, but did not eliminate, mature biofilms, reinforcing its therapeutic promise in combination with antibiotics for biofilm-based infections. Genomic analysis did not identify any known bacterial virulence, toxin, human pathogenicity-associated, or antibiotic-resistance genes, further supporting its suitability as a candidate for phage therapy.

In light of our metagenomic analysis, the differential detection patterns of *C. avidum* and *C. acnes* across the wastewater treatment stages provide context for their environmental occurrence, the challenges associated with isolating *Cutibacterium*-infecting phages [], and the recovery of ΦCA1NRNZ from the First Treatment sampling point [18,19,20]. Wastewater represents a reservoir of human-associated microbial DNA and bacteriophages, and early treatment stages are expected to retain a larger fraction of influent-associated microorganisms and their phages before biological processing. Consistent with this rationale, *C. avidum*-classified reads were detected only at the two earliest stages, the Start Point and First Treatment stages, and at low read counts (51 and 30 reads, respectively). In contrast, no *C. avidum*-classified reads were detected in the downstream Bio-Anaerobic, Bio-Aerobic, or Before HCl stages. Although read classification does not distinguish viable bacteria from host-derived DNA, the detection of *C. avidum*-classified reads in the early wastewater samples supports the rationale for targeting these stages for *C. avidum* phage isolation. This restricted detection pattern contrasted with that of *C. acnes*, which was detected across all five treatment stages and at higher classified-read counts, with the highest classified-read count (2678 reads) observed at the final “Before HCl” point. This difference suggests that closely related *Cutibacterium* species may differ in their abundance, persistence, or detectability during wastewater treatment, despite their phylogenetic relatedness [18,19,20]. One possible explanation is that *C. avidum* may be less tolerant than *C. acnes* to the competitive microbial environment or metabolic stresses encountered during secondary wastewater treatment. However, this interpretation requires confirmation beyond read-count data. Furthermore, the increase in *C. acnes* reads at the final treatment point could reflect niche stability, aggregation that bypasses standard removal processes, or differential DNA persistence or release at this stage.

The absence of detectable *C. avidum*-classified reads from the downstream biological treatment stages further supports the choice of early-stage wastewater samples as a suitable sampling window for recovering *C. avidum*-infecting phages. If *C. avidum* or *C. avidum*-derived DNA is detectable only at low abundance and transiently during wastewater treatment, its infecting phages may also be relatively rare and more likely to be recovered from early-stage samples. This context may also help explain why multiple independent wastewater collection attempts were required before ΦCA1NRNZ was isolated.

Taken together, our findings provide, to the best of our knowledge, the first description of a lytic phage infecting *C. avidum*. Within the tested panel, ΦCA1NRNZ lysed all 11 clinical *C. avidum* isolates and showed no activity against any of the 25 *C. acnes* strains tested, indicating a broad but species-restricted host range. In addition, ΦCA1NRNZ demonstrated significant in vitro anti-biofilm activity.

These findings should be interpreted in light of several limitations. This study characterizes a single phage isolate obtained from a single wastewater facility and tested against a limited clinical isolate collection. In addition, the antibacterial experiments did not include an antibiotic comparator, an antibiotic-combination arm, or a non-target control phage in the biofilm assays. The observed antibiofilm effect, although significant, represented a reduction rather than complete eradication of mature biofilms.

Nonetheless, ΦCA1NRNZ provides a foundation for further development of *C. avidum*-targeting phage approaches. Given its narrow host range, it may be suitable for further evaluation within personalized phage-matching frameworks and, following additional validation, in compassionate-use treatment of refractory *C. avidum*. Essential next steps include in vivo testing in prosthetic device infection models, evaluation of synergy with antibiotics, formal safety assessment, and structural characterization of the receptor-binding proteins underlying its host specificity. Such studies will be needed to determine the clinical applicability of ΦCA1NRNZ and may offer a rational basis for engineering expanded-spectrum *Cutibacterium*-targeting phage variants.

## 4. Materials and Methods

### 4.1. Bacterial Strains and Identification

*Cutibacterium avidum* and *Cutibacterium acnes* strains were isolated from acne lesions of patients (>10 years old) at Hadassah–Hebrew University Medical Center, under ethics approval (HMO-445-22) [12,21]. Lesions were disinfected with 70% isopropanol, and material was extracted by manual expression or sterile needle puncture. Samples were collected with sterile swabs (Copan ESwab^®^, Brescia, Italy) and streaked on Wilkins–Chalgren (Oxoid, Hampshire, UK)agar with furazolidone to inhibit staylococci. Plates were incubated anaerobically at 37 °C for up to 7 days. Isolates were purified by streaking, examined microscopically, and identified by MALDI-TOF (bioMerieux, Marcy I’Etolie, France). Unidentified strains underwent 16S *rRNA* sequencing, with BLAST+ (version 2.17.0) based species identification.

### 4.2. Phage Isolation, Amplification, and Storage

Phage ΦCA1NRNZ was isolated from wastewater collected at the Sorek Wastewater Treatment Facility (Jerusalem, Israel). The sample was centrifuged (Eppendorf 5430R, Hamburg, Germany) at 5000× *g* for 10 min, and the supernatant was passed through a 0.22 µm filter. Five milliliters of filtrate were incubated with *Cutibacterium avidum* strain 48 as host strain in Wilkins–Chalgren broth) under anaerobic conditions at 37 °C for 48 h. After incubation, the culture was centrifuged (5000× *g*, 10 min) and filtered again (0.22 µm). Ten microliters of the filtrate were spotted onto a lawn of *C. avidum* 48 and incubated anaerobically [12].

A single plaque was streak-purified on fresh *C. avidum* lawns through five consecutive passages [12]. The purified phage was propagated in Wilkins–Chalgren medium with *C. avidum* 48 until a titer of 10^9^ PFU/mL was achieved. For long-term storage, the phage was incubated with the host strain for 30 min, centrifuged (5000× *g*, 10 min), and the pellet resuspended in Wilkins–Chalgren broth containing 25% glycerol. The suspension was stored at −80 °C.

### 4.3. Plaque Assay

Plaque assays were performed according to IPTC protocols [8]. Fresh *C. avidum* cultures were adjusted to OD_600_ = 1.0 in Wilkins–Chalgren broth. One hundred microliters of bacterial suspension were mixed with 3.5 mL of pre-warmed 0.3% agarose and overlaid onto Wilkins–Chalgren agar plates. For titer determination, six 10-fold serial dilutions of phage were prepared, and 10 µL of each dilution was spotted onto the lawn. Host range was assessed by spotting 10 µL of undiluted phage suspension. Plates were incubated aerobically or anaerobically at 37 °C for 24 h.

### 4.4. Transmission Electron Microscopy

Phage morphology was examined by TEM following established protocols [12]. Briefly, 1 mL of phage suspension (10^9^ PFU/mL) was centrifuged at 20,000× *g* for 2 h at 25 °C (Witeg Labortechnik GmbH, Wertheim, Germany). The pellet was resuspended in 200 µL of 5 mM MgSO_4_. Ten microliters were applied to carbon-coated copper grids, stained with 2% uranyl acetate for 1 min, and imaged using a JEM-1400 Plus electron microscope (JEOL, Tokyo, Japan) with a Gatan Orius 600 CCD camera (Gatan Inc., Pleasanton, CA, USA).

### 4.5. Phage DNA Extraction, Sequencing, and Bioinformatics

Phage DNA was extracted with the Norgen Biotek Phage DNA Isolation Kit (Thorold, ON, Canada). Libraries were prepared and sequenced on an Illumina NextSeq 500 (Illumina Inc., San Diego, CA, USA) platform (single-end, 150 bp), achieving >200× coverage. Reads were trimmed with BBDuk (adapter removal, Q < 5 trimming, reads <60% length discarded, low-complexity trimming with entropy ≥ 0.7, sliding window of 50 bases, k-mer size = 5) and assembled using SPAdes (default settings). Contigs were refined in Geneious Prime v2024.0.3 (highest sensitivity).

Annotation used Pharokka v1.7 and Phold v2.1, with CDS prediction via PHANOTATE [22]. *tRNA* and *tmRNA* genes were detected with tRNAscan-SE 2.0 and Aragorn. CRISPR arrays were identified using CRT. Sequence homology and function of proteins were based on PHROGs (https://phrogs.lmge.uca.fr/, accessed on 4 June 2026) using MMseqs2 (Release 18-8cc5c) and PyHMMER [23].

Phage termini and packaging mode were characterized using PhageTermVirome (version 4.3, https://pypi.org/project/phagetermvirome/, accessed on 4 June 2026). Lytic/Temperate nature was evaluated using PhaBOX v2.1.13 (https://phage.ee.cityu.edu.hk/, accessed on 4 June 2026), and taxonomy was assigned using taxMyPhage [24]. Virulence-factor analysis was performed using Abricate with all databases (https://github.com/tseemann/abricate, accessed on 4 June 2026). Nucleotide comparisons were performed via BLAST+ (version 2.17.0) [25]. The phage genome was deposited in GenBank (accession PV441878.1).

### 4.6. Growth Curve Analysis

Growth curve assays were performed according to IPTC protocols [7]. Briefly, overnight cultures of *C. avidum* were adjusted to OD_600_ = 1.0 in Wilkins–Chalgren broth and then diluted 1:100. Phage stocks were titrated by plaque assay one day before each experiment and diluted to achieve a target MOI of approximately 1 in 96-well plates. Plates were incubated at 37 °C under aerobic or anaerobic conditions, and OD_600_ was measured every 20 min for 24 h using a BioTek Synergy H1 plate reader (BioTek Instruments Inc., Winooski, VT, USA), with 5 s of shaking before each read. Monitoring was not extended beyond 24 h.

### 4.7. Biofilm Crystal Violet Assay

Biofilms were formed in 96-well plates by incubating 200 µL of *C. avidum* suspension (10^8^ CFU/mL in Wilkins–Chalgren medium) under anaerobic conditions at 37 °C for 48 h [25]. Biofilm was treated with either phage or control for a period of 24 h, then non-adherent cells were removed by washing with 0.9% NaCl, and the adherent biofilm was stained with 0.1% crystal violet (CV) (Sigma-Aldrich, St. Louis, MO, USA) for 15 min at room temperature. Wells were washed twice with water, air-dried, and CV solubilized with 100 µL of 30% acetic acid. Absorbance at 550 nm was measured in a microplate reader [26].

### 4.8. Extracellular ATP Quantification

Biofilms were prepared and treated as described above. Ten microliters of phage suspension or saline were added to each well (final volume: 75 µL). The RealTime-Glo™ Extracellular ATP Assay (Promega, Madison, WI, USA) was used per manufacturer’s instructions, adding 25 µL reagent/well. Luminescence was measured every 20 min in BioTek Synergy H1 plate reader using Gen5 2.07 [26,27,28].

### 4.9. Colony-Forming Unit (CFU) from Biofilm

Biofilms were washed with saline, mechanically disrupted, and sonicated for 5 min (SONOREX, Bandelin, Berlin, Germany). Ten-fold dilutions were plated on LB agar, incubated at 37 °C for 24 h, and colonies counted (CFU/mL) [26,29].

### 4.10. Bacterial Sewage Abundance

Wastewater samples (0.5 L) were collected from five sequential treatment stages at the Sorek Wastewater Treatment Facility (Jerusalem, Israel): Start Point, First Treatment, Bio-Anaerobic, Bio-Aerobic, and Before HCl. From each collected sample, a 0.2 mL aliquot was processed for DNA extraction. Total genomic DNA was extracted using the Microbiome DNA Isolation Kit (Product #64100, Norgen Biotek Corp., Thorold, ON, Canada) following the manufacturer’s instructions. Briefly, samples were treated with Lysis Buffer E and Lysis Additive A, then incubated at 65 °C for 5 min to ensure efficient cell lysis. The lysate was then clarified by centrifugation at 20,000× *g* and further purified using Binding Buffer I, incubated on ice. After the addition of 70% ethanol, the mixture was loaded onto a spin column and centrifuged at 10,000× *g*. The column-bound DNA was washed sequentially with Binding Buffer B and Wash Solution A to remove protein and salt contaminants. Finally, the purified DNA was eluted using Elution Buffer B and stored at −20 °C for further sequencing. Sequencing libraries were constructed using the Nextera XT DNA Library Preparation kit (FC-131-1096, Illumina Inc., San Diego, CA, USA) according to the manufacturer’s protocol, with reagent and input DNA volumes reduced to half. Samples were then sequenced on Illumina NextSeq 500 platform using single-end 150 bp reads. The bioinformatic analysis, including read trimming and classification, was performed using the BV-BRC platform (https://www.bv-brc.org/, accessed on 4 June 2026). The abundance of *Cutibacterium acnes* and *Cutibacterium avidum* was extracted from the Kraken2/Bracken classification output for each of the five sewage treatment points (Start Point, First Treatment, Bio-Anaerobic, Bio-Aerobic, and Before HCl). Read counts (number of fragments covered) were retrieved at the species level (NCBI Taxonomy IDs 399,497 and 1,031,709, respectively) and visualized using a grouped bar plot in R v4.x with ggplot2 V4.0.2.

### 4.11. Statistical Analysis and Graph Preparation

All statistical evaluations and graph generation were performed using GraphPad Prism version 8.0.2 (GraphPad Software, La Jolla, CA, USA). Comparisons between groups were carried out with a two-tailed unpaired Student’s *t*-test, considering *p* < 0.05 as statistically significant. For the CFU viability analysis shown in Figure 6C, CFU/mL values were log10-transformed before statistical comparison, and the two-tailed unpaired Student’s *t*-test was performed on the log10-transformed values. Data are presented as the mean values obtained from three independent experiments, with error bars indicating the standard deviation (±SD).

### 4.12. Data Availability

All data supporting the findings of this manuscript are available from the corresponding author upon reasonable request. The genome sequence of phage ΦCA1NRNZ has been deposited in GenBank under accession number PV441878.1 (https://www.ncbi.nlm.nih.gov/nuccore/PV441878.1, accessed on 4 June 2026).

## Data Availability

All new data created or analyzed in this study are available in public databases. A new isolated phage genome is available at GenBank: https://www.ncbi.nlm.nih.gov/nuccore/PV441878.1 (accessed on 4 June 2026).

## Supplementary Materials

The following supporting information can be downloaded at: https://www.mdpi.com/article/10.3390/antibiotics15070659/s1, Table S1. Genome annotation of ΦCA1NRNZ. Predicted coding sequences (CDSs) of the phage genome with their corresponding genomic coordinates, lengths, and transcriptional orientation. Table S2. Efficiency of plating (EOP) of the isolated phage on different *Cutibacterium avidum* strains. The EOP was calculated by dividing the phage titer obtained on each *C. avidum* strain by the phage titer on the reference strain *C. avidum* 48. For all tested strains, plaques appeared clear with a turbid expanding halo.
