# Supplementary material for: Isolation and Characterization of ΦCA1NRNZ, a Lytic Bacteriophage Targeting the Emerging Device-Associated Pathogen Cutibacterium avidum
Source: Antibiotics (Basel). 2026 Jul 3;15(7):659. doi: 10.3390/antibiotics15070659 (PMC13405974; doi:10.3390/antibiotics15070659)
Supplement: Supplementary file 1 [file antibiotics-15-00659-s001.zip › antibiotics-4388751-supplementary.pdf]

Supplementary Material

Table S1. Genome annotation of ΦCA1NRNZ. Predicted coding sequences (CDSs) of the phage genome with their corresponding genomic coordinates, lengths, and transcriptional orientation.

Name	*Minimum*	*Maximum*	*Length*	*Direction*
collagen like minor tail CDS	16752	17351	600	forward
dATP / dGTP pyrophosphohydrolase CDS	26025	26372	348	reverse
DNA methyltransferase CDS	32370	33323	954	forward
DNA primase CDS	22068	22730	663	reverse
DnaB-like replicative helicase CDS	23324	24166	843	reverse
E3 ubiquitin-protein ligase SopA CDS	26460	27149	690	reverse
E3 ubiquitin-protein ligase SopA CDS	29068	29607	540	reverse
exonuclease CDS	25130	26113	984	reverse
exonuclease CDS	20247	21218	972	reverse
head closure Hc1 CDS	7747	8088	342	forward
head maturation protease CDS	4851	5600	750	forward
head scaffolding protein CDS	5654	6268	615	forward
head-tail adaptor CDS	7282	7743	462	forward
HNH endonuclease CDS	33368	33667	300	forward
holin CDS	18359	18721	363	forward
HTH DNA binding domain protein CDS	21271	21447	177	reverse
hypothetical protein CDS	8337	9374	1038	reverse
hypothetical protein CDS	17409	18311	903	reverse
hypothetical protein CDS	21456	22031	576	reverse
hypothetical protein CDS	24675	25220	546	reverse
hypothetical protein CDS	30181	30717	537	reverse
hypothetical protein CDS	24191	24628	438	reverse
hypothetical protein CDS	18721	19143	423	forward
hypothetical protein CDS	19264	19677	414	reverse
hypothetical protein CDS	28639	29046	408	reverse
hypothetical protein CDS	29712	30101	390	reverse
hypothetical protein CDS	350	712	363	reverse
hypothetical protein CDS	16157	16486	330	forward
hypothetical protein CDS	19921	20250	330	reverse
hypothetical protein CDS	27202	27516	315	reverse
hypothetical protein CDS	28341	28652	312	reverse
hypothetical protein CDS	9824	10111	288	forward
hypothetical protein CDS	30931	31182	252	forward
hypothetical protein CDS	31464	31694	231	reverse
hypothetical protein CDS	87	254	168	reverse
hypothetical protein CDS	9431	9580	150	reverse
hypothetical protein CDS	31912	32061	150	reverse
hypothetical protein CDS	32072	32221	150	reverse
hypothetical protein CDS	28228	28341	114	reverse

hypothetical protein CDS	9741	9851	111	forward
hypothetical protein CDS	22682	22792	111	reverse
hypothetical protein CDS	1514	1621	108	forward
hypothetical protein CDS	19187	19294	108	forward
hypothetical protein CDS	30726	30833	108	reverse
hypothetical protein CDS	31288	31383	96	forward
hypothetical protein CDS	30840	30929	90	forward
lectin CDS	15342	16103	762	forward
major head protein CDS	6276	7235	960	forward
minor tail protein CDS	14021	15232	1212	forward
minor tail protein CDS	13195	14010	816	forward
minor tail protein CDS	19628	19927	300	reverse
minor tail protein CDS	16508	16765	258	forward
neck protein CDS	8170	8379	210	forward
phosphoadenosine phosphosulfate reductase CDS	714	1454	741	reverse
portal protein CDS	3527	4846	1320	forward
RusA-like Holliday junction resolvase CDS	22931	23302	372	reverse
tail assembly chaperone CDS	9588	9725	138	forward
tail length tape measure protein CDS	10116	13067	2952	forward
terminase large subunit CDS	2100	3530	1431	forward
terminase small subunit CDS	1694	2026	333	forward
predicted phage fitness factor CDS	27473	28231	759	reverse

Table S2. Efficiency of plating (EOP) of the isolated phage on different Cutibacterium avidum strains. The EOP was calculated by dividing the phage titer obtained on each C. avidum strain by the phage titer on the reference strain C. avidum 48. For all tested strains, plaques appeared clear with a turbid expanding halo**.**

Bacterial strain	EOP
***C. avidum* 4**	**2.508**
***C. avidum* 13C**	**1.13**
***C. avidum* 16**	**5.55**
***C. avidum* 20**	**1.12**
***C. avidum* 21**	**0.92**
***C. avidum* 25**	**2.41**
***C. avidum* 33**	**1.45**
***C. avidum* 34**	**1.25**
***C. avidum* 42**	**0.75**
***C. avidum* 46**	**3.60**
***C. avidum* 48**	**1.0**
